# Inhibition of the 3-mercaptopyruvate sulfurtransferase—hydrogen sulfide system promotes cellular lipid accumulation

**DOI:** 10.1007/s11357-022-00600-9

**Published:** 2022-06-10

**Authors:** Giovanna Casili, Elisa Randi, Theodora Panagaki, Karim Zuhra, Maria Petrosino, Csaba Szabo

**Affiliations:** Chair of Pharmacology, Faculty of Science and Medicine, University of Fribourg, Chemin du Musée 18, 1700 Fribourg, Switzerland

**Keywords:** Adipocytes, Hydrogen sulfide, 3-Mercaptopyruvate sulfurtransferase, Obesity

## Abstract

H_2_S is generated in the adipose tissue by cystathionine γ-lyase, cystathionine β-synthase, and 3-mercaptopyruvate sulfurtransferase (3-MST). H_2_S plays multiple roles in the regulation of various metabolic processes, including insulin resistance. H_2_S biosynthesis also occurs in adipocytes. Aging is known to be associated with a decline in H_2_S. Therefore, the question arises whether endogenous H_2_S deficiency may affect the process of adipocyte maturation and lipid accumulation. Among the three H_2_S-generating enzymes, the role of 3-MST is the least understood in adipocytes. Here we tested the effect of the 3-MST inhibitor 2-[(4-hydroxy-6-methylpyrimidin-2-yl)sulfanyl]-1-(naphthalen-1-yl)ethan-1-one (HMPSNE) and the H_2_S donor (GYY4137) on the differentiation and adipogenesis of the adipocyte-like cells 3T3-L1 in vitro. 3T3-L1 cells were differentiated into mature adipocytes in the presence of GYY4137 or HMPSNE. HMPSNE significantly enhanced lipid accumulation into the maturing adipocytes. On the other hand, suppressed lipid accumulation was observed in cells treated with the H_2_S donor. 3-MST inhibition increased, while H_2_S donation suppressed the expression of various H_2_S-producing enzymes during adipocyte differentiation. 3-MST knockdown also facilitated adipocytic differentiation and lipid uptake. The underlying mechanisms may involve impairment of oxidative phosphorylation and fatty acid oxidation as well as the activation of various differentiation-associated transcription factors. Thus, the 3-MST/H_2_S system plays a tonic role in suppressing lipid accumulation and limiting the differentiation of adipocytes. Stimulation of 3-MST activity or supplementation of H_2_S—which has been recently linked to various experimental therapeutic approaches during aging—may be a potential experimental approach to counteract adipogenesis.

## Introduction

Hydrogen sulfide (H_2_S) is an endogenous gaseous mediator produced with multiple regulatory roles in health and disease. Previous studies have shown that H_2_S regulates, among others, cardiovascular function, inflammation, insulin resistance, and glucose metabolism [1–6]. In mammals, H_2_S is mainly synthetized by three enzymes: cystathionine-β-synthase (CBS) and cystathionine-γ-lyase (CSE) (both localized in the cytosol) and 3-mercaptopyruvate sulfurtransferase (3-MST) (localized both in cytosol and mitochondria), with additional contribution from other, less characterized enzyme systems (e.g., d-amino acid oxidase) and non-enzymatic reactions [6]. H_2_S levels are declining during aging due to a combination of decreased production and/or increased consumption of this mediator [6, 7]. Since aging and obesity are closely interlinked [8], the question arises as to whether a suppression of endogenous H_2_S generation may modulate adipocyte fat accumulation, maturation, and the development of obesity.

Adipogenesis is a tightly controlled multi-step process, which leads to the formation of new adipocytes from their precursor stem cells. Adipogenesis-related molecules include fatty acid–binding protein 4, peroxisome proliferator–activated receptor γ (PPARγ), CCAAT/enhancer-binding protein α, sterol regulatory element–binding protein-1, carbohydrate responsive element–binding protein, fatty acid synthase, adiponectin, hormone-sensitive lipase, and perilipin A [9]. All three H_2_S-producing enzymes CBS, CSE, and 3-MST are endogenously expressed in adipocytes [10–16]. So far, the majority of studies have focused on the role of CSE and demonstrated that genetic and pharmacological inhibition of CSE significantly suppresses adipogenesis in 3T3-L1 cells [10–14]. However—and in line with the well-known biphasic or bell-shaped concentration response of H_2_S—other studies suggested that H_2_S donors can also suppress adipogenesis in the same model system [17].

The potential contribution of the 3-MST/H_2_S system to the process of adipogenesis is incompletely understood. Morton et al. was the first to identify *Mpst* as a potential candidate gene associated to obesity, using a bioinformatic-based approach [18]. Afterwards, it has been established a link between reduced 3-MST expression and obesity in mice fed with a high-fat diet, as well as in genetically obese (db/db) mice that lack the leptin receptor [19]. More recently, it has been reported that 3-MST is expressed in the adipose tissue, both in pre-adipocytes and in mature adipocytes [19]. These findings led us to investigate the molecular basis of the involvement of 3-MST in cellular lipid accumulation during adipogenesis.

We have used a well-characterized experimental model (differentiation and adipogenesis of the adipocyte-like cells 3T3-L1 in vitro) and inguinal white adipose tissue (iWAT) from C57Bl/6 J mice, as an ex vivo model. We have investigated the role of 3-MST/H_2_S pathway on adipogenesis, using either a genetic approach (3-MST silencing) or a pharmacological approach which utilized the novel, selective 3-MST inhibitor (2-[(4-hydroxy-6-methylpyrimidin-2-yl)sulfanyl]-1-(naphthalen-1-yl)ethan-1-one; HMPSNE) [20–22] or the slow-releasing H_2_S donor (4-methoxyphenyl(morpholino)phosphinodithioate morpholinium salt; GYY4137) [22,b24].

## Materials and methods

### Reagents

The H_2_S donor GYY4137 was purchased from Sigma-Aldrich (St Louis, MO, USA) and was resuspended in cell culture medium. The 3-MST inhibitor HMPSNE (MolPort, Riga, Latvia) was dissolved in DMSO (Sigma-Aldrich) immediately before adding it to the culture medium. Oil Red O was purchased from Sigma-Aldrich (O-0625). 3-(4,5-dimethylthiazol-2-yl)-2,5-Diphenyltetrazoliumbromide (Sigma-Aldrich) for the MTT assay was dissolved in PBS. LDH release was measured using the cytotoxicity detection kit plus (Roche, Mannheim, Germany). Intracellular H_2_S was detected with the H_2_S sensing probe AzMC (7-azido-4-methylcoumarin) (Sigma-Aldrich). For the analysis of transcription factor activation, the Oxidative Stress TF Activation Profiling Plate Array was purchased from Signosis (Santa Clara, CA, USA). Insulin and dexamethasone for cell differentiation process were purchased from Sigma-Aldrich.

### Cell culture and treatments

Murine 3T3-L1 pre-adipocytes were cultured in Advanced DMEM/F12 (Gibco, Thermo Fisher Scientific) supplemented with 10% fetal bovine serum (FBS, Gibco, Thermo Fisher Scientific), GlutaMAX (Gibco, Thermo Fisher Scientific), penicillin (100 µg/ml), and streptomycin (100 µg/ml) (Invitrogen) in a humidified atmosphere of 5% CO_2_ at 37 °C. To induce differentiation in adipocytes, normal growth medium was substituted with *differentiation medium* (Advanced DMEM/F12 supplemented with 1 µg/ml insulin, 1 µg/ml dexamethasone and 10% FBS). After 2 days, *differentiation medium* was replaced with *maintenance medium* (Advanced DMEM/F12, supplemented with 10% FBS and 1 µg/ml insulin) and changed every 2 days up to 1 week. Cell treatments were performed during the differentiation process, by adding different concentrations GYY4137 (1, 3, and 6 mM) or HMPSNE (30 and 100 µM) to both differentiation and maintaining media on day 1 and day 5.

### Measurement of cellular metabolic activity and cell viability

The 3T3-L1 pre-adipocytes were seeded at a density of 5 × 10^3^ cells per well in 96-well plates and subjected to cell differentiation and treatments. For mitochondrial activity measurements, adipocytes were incubated with 0.5 µg/ml MTT for 3 h at 37 °C in the dark. After medium removal, DMSO was added to each well, and the plates were shaken to dissolve the formazan crystals. Absorbance was measured at 590 nm using a multiwell plate reader (Infinite 200Pro, Tecan, Männedorf, Switzerland) [21, 22]. The percentage of MTT conversion was calculated by defining the control samples as 100%.

Cell viability (the effect of various treatments on the release of lactate dehydrogenase (LDH) into the supernatants, an index of cell necrosis) was quantified by measuring the LDH content of the culture medium as described [21, 22]. After adding the LDH mix solution to the supernatants, absorbance was measured at 360–490 nm using a multiwell plate reader (Tecan).

### Oil Red O staining to quantify cellular lipid content

Pre-adipocytes were seeded at a density of 3 × 10^4^ cells per well in 12-well plates. After differentiation and cell treatments, adipocytes were fixed with 10% formalin in PBS for 1 h and washed with 60% isopropanol. Cellular lipid content was quantified using the Oil Red O method as described [8, 10]. After drying at room temperature, cells were stained with Oil Red O solution for 30 min, and were then washed with distilled water. The phenotypic changes of adipogenic differentiation were observed using an inverted phase-contrast microscope (Olympus LX81, Japan). To quantify the amount of Oil Red O–stained lipids, cells were incubated with 100% isopropyl alcohol for 10 min and the absorbance of the supernatants was measured at 500 nm using a plate reader (Tecan).

### Detection of H_2_S with AzMC probe

3T3-L1 pre-adipocytes were seeded at a density of 5 × 10^3^ cells per well in 96-well plates. Cellular H_2_S levels were quantified using live cell imaging as described [8, 21, 22]. After differentiation and treatments, cells were incubated with 10 µM of the H_2_S sensitive probe AzMC in HBSS buffer and further incubated at 37 °C for 1 h. The specific fluorescence of the dye was visualized using a Leica DFC360. FX microscope and images were captured with Leica Application Suite X (LAS X) software (Leica Biosystems Nussloch GmbH, Germany). Images were analyzed with ImageJ software (v. 1.8.0; NIH, Bethesda, Maryland, USA) and data and graphed with GraphPad Prism 8 (GraphPad Software Inc.; San Diego, California, USA).

### Western blotting

Pre-adipocytes were seeded at a density of 6 × 10^4^ cells per well in 6-well plates. Following differentiation and treatments, cells were washed twice with PBS and incubated on ice with lysis buffer (ELISA lysis buffer, Thermo Fisher Scientific) containing proteinase and phosphatase inhibitor (Cell Signaling technology, Leiden, The Netherlands). Proteins were separated by SDS-PAGE, transferred to a nitrocellulose membrane (Thermo Fisher Scientific), and incubated with specific primary antibodies. The antibodies used in this study were directed against β-actin (Cell Signaling Technology, 8H10D10, 1:2,000), CBS (Cell Signaling Technology, D8F2P, 1:500), CSE (Abcam, Ab151769, 1:1,000), 3-MST (Abcam, Ab85377, 1:500), ETHE-1 (Abcam, Ab174302, 1:1,000), TST/rhodanese (Abcam, Ab231248, 1:1,000), and PPAR-γ (CST, 2430S, 1:1,000). Signals from HRP-coupled secondary antibodies were detected with ECL Prime Western Blotting Detection Reagent (Sigma-Aldrich), using a luminescent image analyzer (Fusion FX6, Vilber Lourmat, Marne la Vallée, France) and quantified using the ImageJ software (NIH, Bethesda, MD, USA).

### Generation of stable 3-MST knockdown cell lines

The expression vector encoding short hairpin RNA (shRNA) sequence targeting mouse 3-MST in a pLKO.1-puro plasmid was purchased from Sigma-Aldrich (Ref: NM_138670/TRCN0000111025). A non-targeting shRNA (pKLO.1 puromycin resistance vector; Sigma-Aldrich) was used as negative control (shCTR). Viral particles were produced in HEK293T cells by co-transfection of the respective transfer vector (3 μg) with the packaging plasmids pLP1 (4.2 μg), pLP2 (2 μg), and pVSV-G (2.8 μg, all from Invitrogen) using FuGene (Promega Corporation, Madison, WIS, USA), following the manufacturer’s instruction. After lentiviral-pseudotyped particle infection, 3T3-L1 cells were subjected to puromycin selection (Sigma-Aldrich, 2 µg/ml).

### Determination of cellular bioenergetics from white adipose tissue

C57Bl/6 J mice were purchased from the Jackson Laboratory. 3-MST knockout mice (*Mpst*^*−/−*^), generated on a C57Bl/6 J background, were provided by Professor Noriyuki Nagahara (Nippon Medical School) [25]. Mice were fed with chow diet made of 10% calories from fat, 20% calories from protein, and 70% calories from carbohydrates (Research Diets, D12450K or Ssniff, E157452-04). All experimental procedures that compared WT and *Mpst*^*−/−*^ mice were performed on 22-week-old mice. The inguinal white adipose tissue (iWAT) was collected as described [26]. All animal-related procedures performed were approved by the Swiss Federal Food Safety and Veterinary Office (license no. FR_2020_31).

Mitochondrial function of mouse inguinal white adipose tissue (iWAT) was assessed using both glucose (Mito Stress assay) and palmitate (fatty acid oxidation assay (FAO assay)) as energy substrates. Mito stress assay was performed using DMEM buffer (Agilent-102353–100), supplemented with 1 mM sodium pyruvate, 2 mM GlutaMAX-1TM, 25 mM glucose, and 4 mg/ml of fatty acid free bovine serum albumin. FAO assay was performed in Krebs–Henseleit Buffer (111 mM NaCl, 4.7 mM KCl, 2 mM MgSO_4_, 1.25 mM CaCl_2_, and 1.2 mM NaH_2_PO_4_), supplemented with 5 mM HEPES buffer, 0.5 mM carnitine, and 4 mg/ml of fatty acid free bovine serum albumin. Assays were carried out using a Seahorse XFe-24 flux analyzer (Agilent Technologies, Santa Clara, California, USA) and XFe24 Islet Capture Microplates equipped with nylon mesh inserts (Agilent-103518–100). After collection, white adipose tissue was kept in the assay media at 37 °C until they were processed. Tissues were cut into small pieces (10 to 20 mg) by mean of a McIlwain tissue chopper set at 0.05-mm thickness. Slices were centered onto nylon inserts and covered with 15 µl of a solution made of 1 part of chicken plasma (Sigma-Aldrich-P3266) and 1 part of 100 UN/ml thrombin (Sigma-Aldrich-T7513), premixed immediately before use. This solution allowed to fix slices on the nylon inserts within O_2_-permeable clots, thus overcoming the problem of free-floating tissue. The inserts were then loaded with a Seahorse Capture Screen Insert Tool (Agilent-101135–100) into XFe Islet Capture Microplates containing assay medium. Appropriate wells received 150 µM HMPSNE (3MST inhibitor) (MolPort–Riga, Latvia) or 100 µM of Etomoxir (carnitine palmitoyl transferase-1 inhibitor) (Sigma-Aldrich-E1905). The plate was then incubated in a CO_2_-free incubator at 37 °C for 1 h to allow temperature and pH equilibration. Only in the case of FAO, just prior to stating the assay, wells received 87.5 µl of 1 mM XF Palmitate-BSA FAO substrate (Agilent-102720–100). In all cases, the final assay volume was 500 µl. The assay protocol consisted in 3-min mix, 3-min wait, and 2-min measurement cycles, with measurement of basal values of oxygen consumption rate (OCR) (4 cycles), followed by injection of 50 µM oligomycin (Sigma-Aldrich-O4876), used to evaluate ATP generation rate (7 cycles). Afterward, 25 µM FCCP (Sigma-Aldrich-C2920) was employed to evaluate the maximal mitochondrial respiratory capacity (7 cycles). Finally, 20 μM of rotenone (Sigma-Aldrich-R8875) and antimycin A (Sigma-Aldrich-A8674) were injected to inhibit the electron transport through the complex I and III, respectively, aiming to detect the extra-mitochondrial OCR (7 cycles). Data were analyzed with Wave (v. 2.6; Agilent Technologies, Santa Clara, California, USA) and graphed with GraphPad Prism 8 (GraphPad Software Inc.; San Diego, California, USA).

### Transcription factor Activation Profiling Array

A transcription factor (TF) Activation Profiling Array was used to compare the activities of multiple TFs simultaneously on adipocyte controls, and GYY4137- or HMPSNE-treated cells. Nuclear extracts from adipocytes were mixed with biotin-labeled probes and allowed to form TF/probe complexes. The bound probes were then hybridized on a 96-well plate, where each well is specifically pre-coated with complementary sequences of the probes. The TF activation was assessed with a luminometer (Tecan). TFs were considered differentially activated when presenting readings of |fold change| > 2 and were normalized to the adipocyte control.

### Statistical analysis

Results are presented as mean values ± standard error of the mean (SEM) of at least three independent experiments or representative Western blots of at least three independent determinations, unless indicated otherwise. Statistical analyses were performed with GraphPad Prism 8 (GraphPad Software; San Diego, CA, USA), using Student’s *t*-test, one-way ANOVA, or two-way ANOVA with multiple comparison and Bonferroni post hoc test where appropriate. *p*-values < 0.05 were considered statistically significant.

## Results

### Characterization of adipogenesis markers in 3T3-L1 cells

**Fig. 1:**
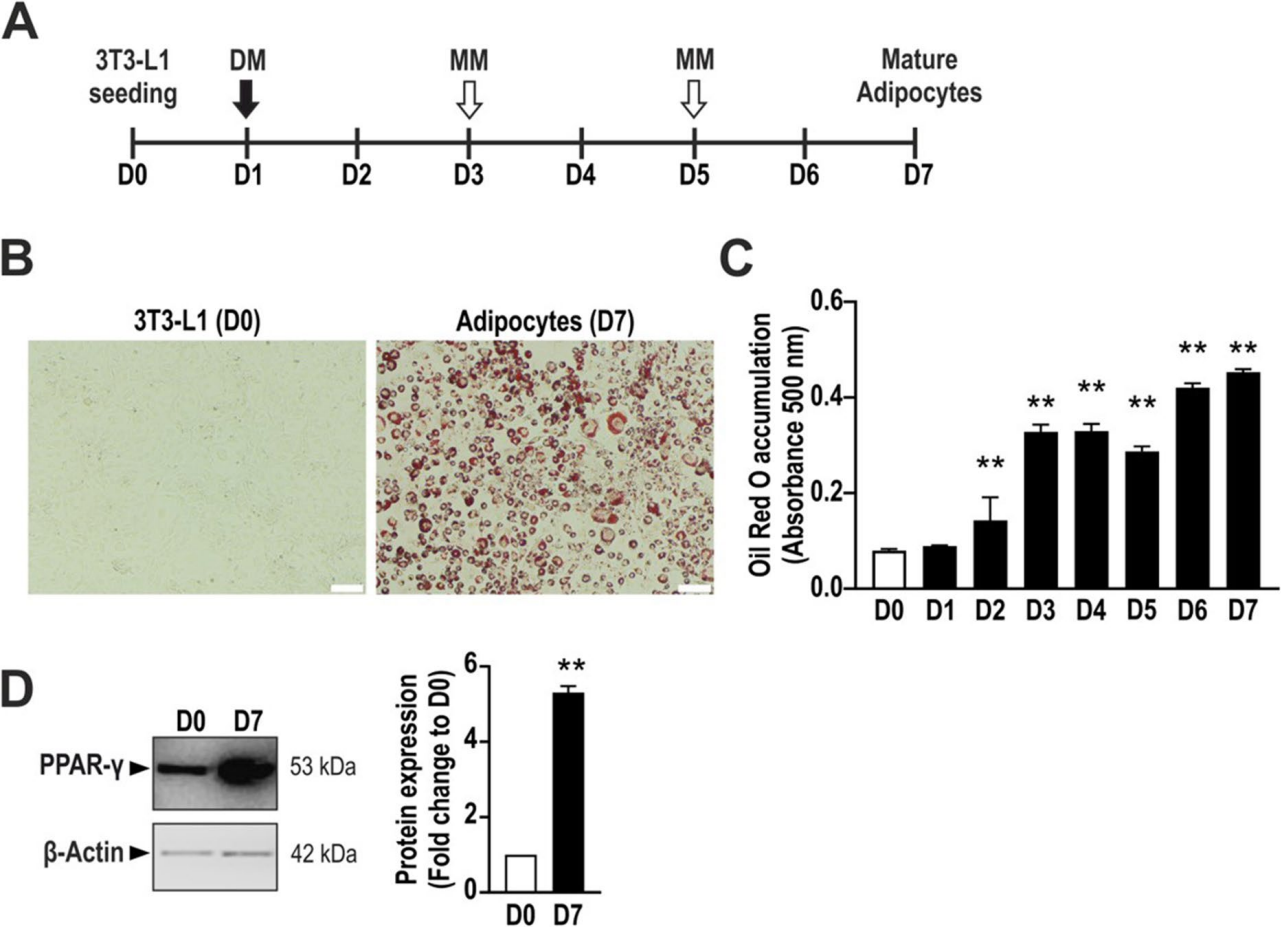
Adipogenesis process in 3T3-L1 cells. **A** Schematic overview of the differentiation process in 3T3-L1 cells. **B** Representative picture showing non-differentiated 3T3-L1 cells (D0) and adipocytes (D7) stained with Oil Red O. **C** Daily quantification of Oil Red O accumulation by measuring its absorbance at 500 nm with a plate reader. **p < 0.01 compared to day 0. **D** Representative immunoblot showing the expression of the adipocyte marker PPAR-γ and its densitometry analysis. β-Actin was used as loading control. D0: day 0 (non-differentiated cells); D7: day 7 (mature adipocytes). DM, differentiation medium; MM, maintenance medium. Data refer to mean values of *N* = 5 independent experiments ± SEM. **p < 0.01 shows significant Oil Red O accumulation on days 2–7 compared to day 0 control or significant PPARγ expression on day 7 compared to day 0 control

Differentiation of 3T3-L1 cells into adipocyte was induced by treating confluent cells with *differentiation medium* on day 1, followed by *maintenance medium* on days 3 and 5, as shown in the scheme (Fig. 1A). Mature adipocytes are characterized by lipid formation and growth arrest. To assess the successful differentiation of 3T3-L1 cells into adipocytes, we evaluate lipid accumulation by staining them with Oil Red O. Differentiated cells displayed a strong Oil Red O signal compared to undifferentiated cells, as assessed by microscope imaging (Fig. 1B). Quantification of cellular Oil Red O content over differentiation showed a time-dependent accumulation of lipids (Fig. 1C). Moreover, the expected increase in the expression of the adipocyte differentiation marker PPAR-γ during adipocyte maturation was confirmed by Western blotting (Fig. 1D).

### Adipocyte lipid accumulation is suppressed by the H_2_S donor GYY4137 and enhanced by the 3-MST inhibitor HMPSNE

**Fig. 2:**
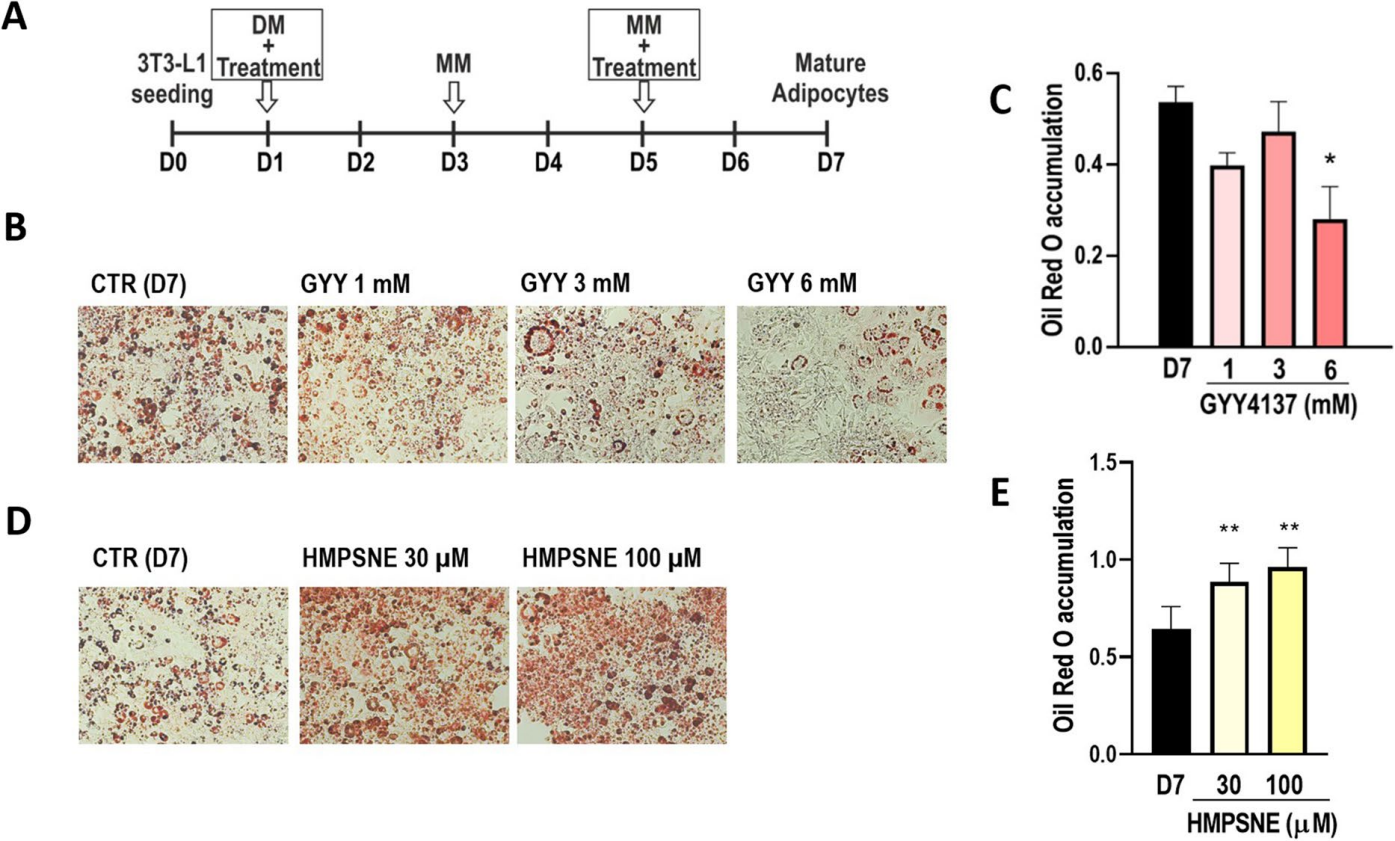
Effects of GYY4137 or HMPSNE treatment on adipogenesis. **A** Scheme of GYY4137 or HMPSNE treatment during adipogenesis. **B** Representative images showing GYY4137-treated adipocytes stained with Oil Red O and **C** corresponding quantification on day 7 (fully differentiated state). **D** Representative images showing HMPSNE-treated adipocytes stained with Oil Red O and **E** corresponding quantification on day 7 (fully differentiated state). Data refer to mean values of *N* = 5 independent experiments ± SEM. *p < 0.05 and **p < 0.01 shows significant effect of HMPSNE or GYY4137 on Oil Red O accumulation on day 7 compared to control values in the absence of pharmacological modulator treatment

To investigate the role of 3-MST in the modulation of lipid accumulation during adipogenesis, 3T3-L1 cells were differentiated in the presence or absence of the H_2_S donor GYY4137 or the 3-MST inhibitor HMPSNE according to the scheme presented in Fig. 2A. GYY4137 was employed at 1 mM, 3 mM, or 6 mM, accounting for a constant H_2_S release during the differentiation process of 40 µM, 80 µM, and 100 µM, respectively, as measured by means of ArrowH_2_S™ Complete Hydrogen Sulfide Measurement System (Lazar Research Labs, Inc., Los Angeles, CA, USA). GYY4137 significantly inhibited adipocyte differentiation, as shown by Oil Red O staining (Fig. 2B, C). Conversely, treatment with HMPSNE promoted adipogenesis as displayed by Oil Red O staining (Fig. 2D, E).

### Effect of GYY4137 and HMPSNE on the viability of differentiating adipocytes

**Fig. 3:**
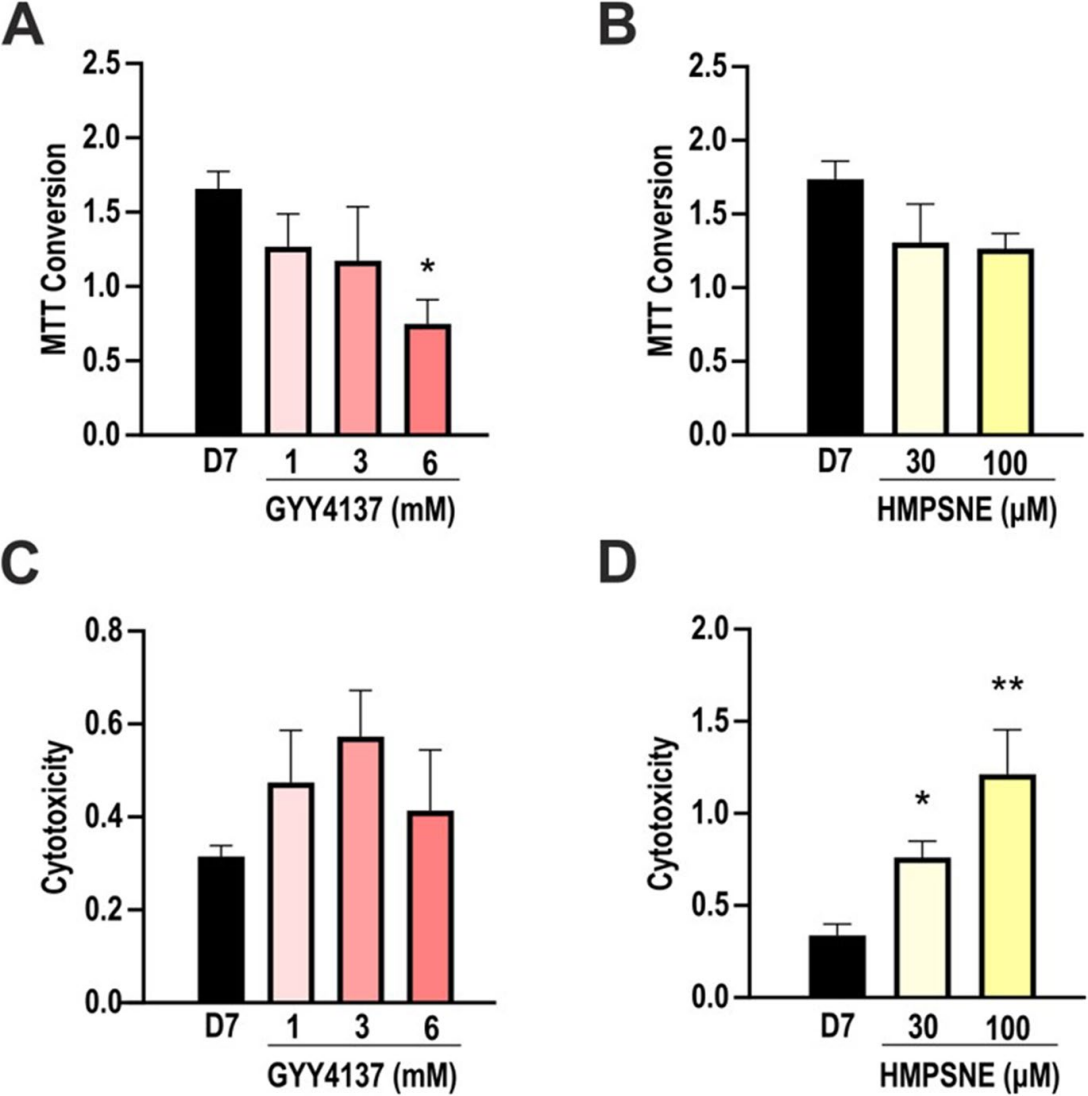
Effects of GYY4137 and HMPSNE on MTT conversion and LDH release, indices of mitochondrial activity and cell viability. **A** Analysis of MTT conversion ability in GYY4137-treated (1, 3, and 6 mM) and **B** HMPSNE-treated (30, 100 µM) cells after differentiation (day 7). **C** LDH assay in GYY4137 and **D** HMPSNE-treated cells after differentiation (day 7). Data refer to mean values of *N* = 5 independent experiments ± SEM. *p < 0.05 and **p < 0.01 show significant effects of GYY4137 or HMPSNE on MTT or LDH values compared to control values in the absence of pharmacological modulator treatment

The reduction of tetrazolium dye MTT to formazan was measured to assess cellular mitochondrial activity. Cells treated with 6 mM GYY4137 showed a significant reduction of MTT conversion ability compared to control (Fig. 3A), while HMPSNE did not show any effect on MTT conversion at the tested concentrations (Fig. 3B).

LDH levels in the supernatant were slightly increased in adipocytes treated with HMPSNE (Fig. 3D), perhaps due to a 3-MST-independent, slight cytotoxicity of this compound at the concentration used. Such effects are cell-type dependent; they also occur in various cancer cell lines as well, although typically at higher (200–300 µM) concentrations [21, 22]. Pharmacological agents’ non-specific effects on cell viability can be limitations of pharmacological experiments; however, currently, HMPSNE is the only specific and cell-permeable inhibitor that is useful to inhibit 3-MST [5], and supplementary experiments with other 3-MST inhibitor classes are not feasible. However, we have conducted experiments using 3-MST silencing as well; the data using this system have provided a confirmation of the effects obtained with the inhibitor (see below).

LDH levels in the supernatant showed no significant differences in GYY4137-treated cells, although a trend for a bell-shaped concentration–response was noted (Fig. 3C).

### Effects of GYY4137 on the expression of various H_2_S-producing and H_2_S-metabolizing enzymes in differentiated adipocytes

**Fig. 4:**
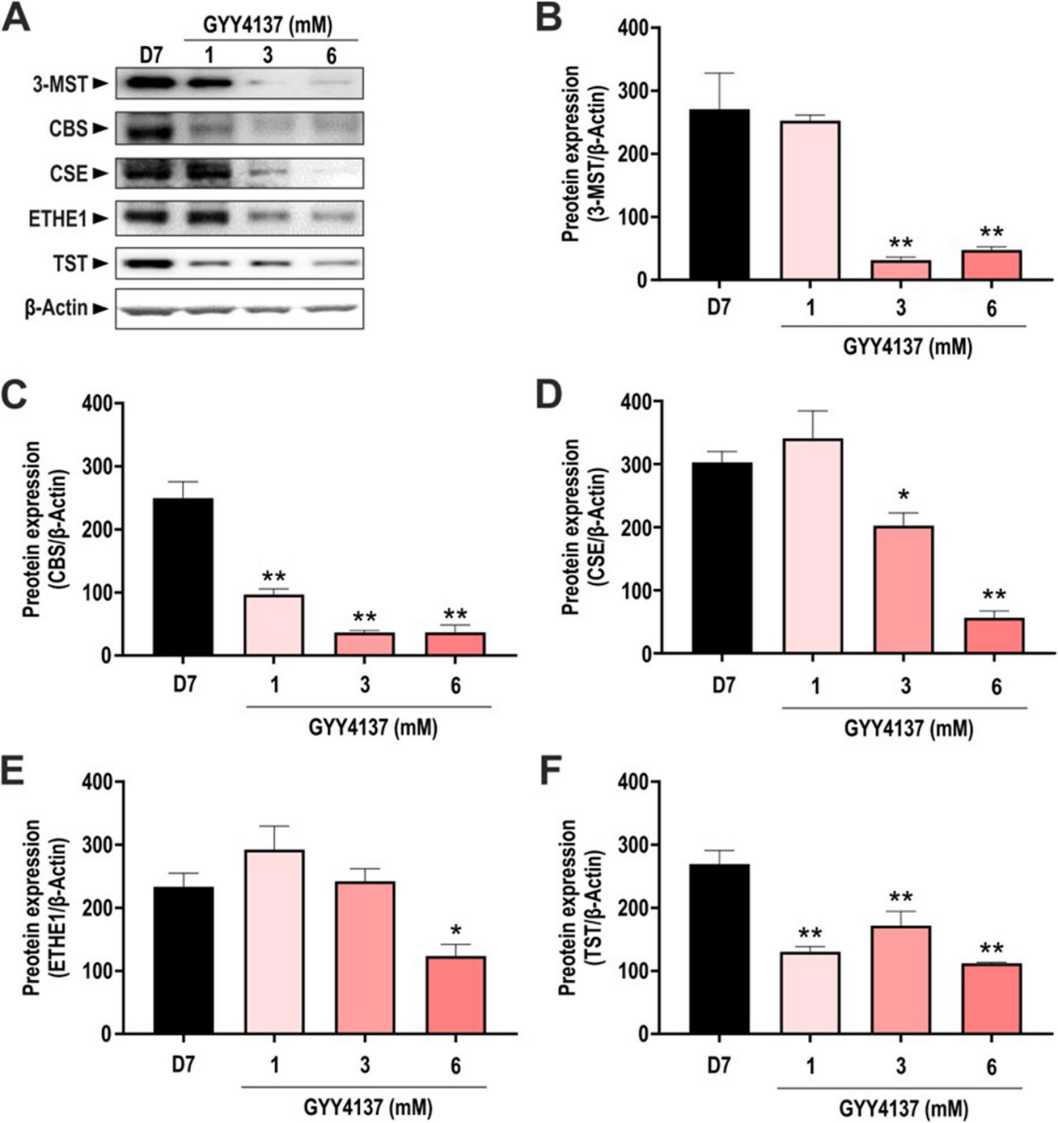
Expression of H_2_S-producing and -catabolizing enzymes in GYY4137-treated cells. **A** Representative Western blot images of adipocytes treated with different concentration of GYY4137 and corresponding densitometry analysis of **B** 3-MST, **C** CBS, **D** CSE, **E** ETHE1, and **F** TST in differentiated adipocytes (day 7). β-Actin was used as loading control. Data refer to mean values of *N* = 5 independent experiments ± SEM. *p < 0.05 and **p < 0.01 show significant differences compared to control values in the absence of GYY417 treatment

Differentiation of 3T3-L1 cells into adipocytes induces the upregulation of all three principal H_2_S-producing enzymes [8, 10]. Indeed, differentiated adipocytes expressed CBS, CSE, and 3-MST, as well as the H_2_S-degrading enzymes ETHE1 and TST (Fig. 4A). To investigate whether GYY4137 treatment was associated with alterations in H_2_S-producing or catabolizing enzymes, the effect of this H_2_S donor was assessed on the expression of 3-MST, CBS, CSE, ETHE1, and TST. 3-MST, CBS, and CSE were all downregulated in the GYY4137-treated cells particularly at 3 mM and 6 mM (Fig. 4A–D). Similarly, TST expression was reduced in GYY4137-treated adipocytes compared to non-treated cells (Fig. 4D), while ETHE-1 levels were downregulated only at 6 mM (Fig. 4E). These results show that GYY4137 (which generates H_2_S levels in the low micromolar concentration range in the current experimental system) downregulates all 3 H_2_S-producing pathways investigated.

### Effects of HMPSNE on the expression of various H_2_S-producing and H_2_S-metabolizing enzymes in differentiated adipocytes

**Fig. 5:**
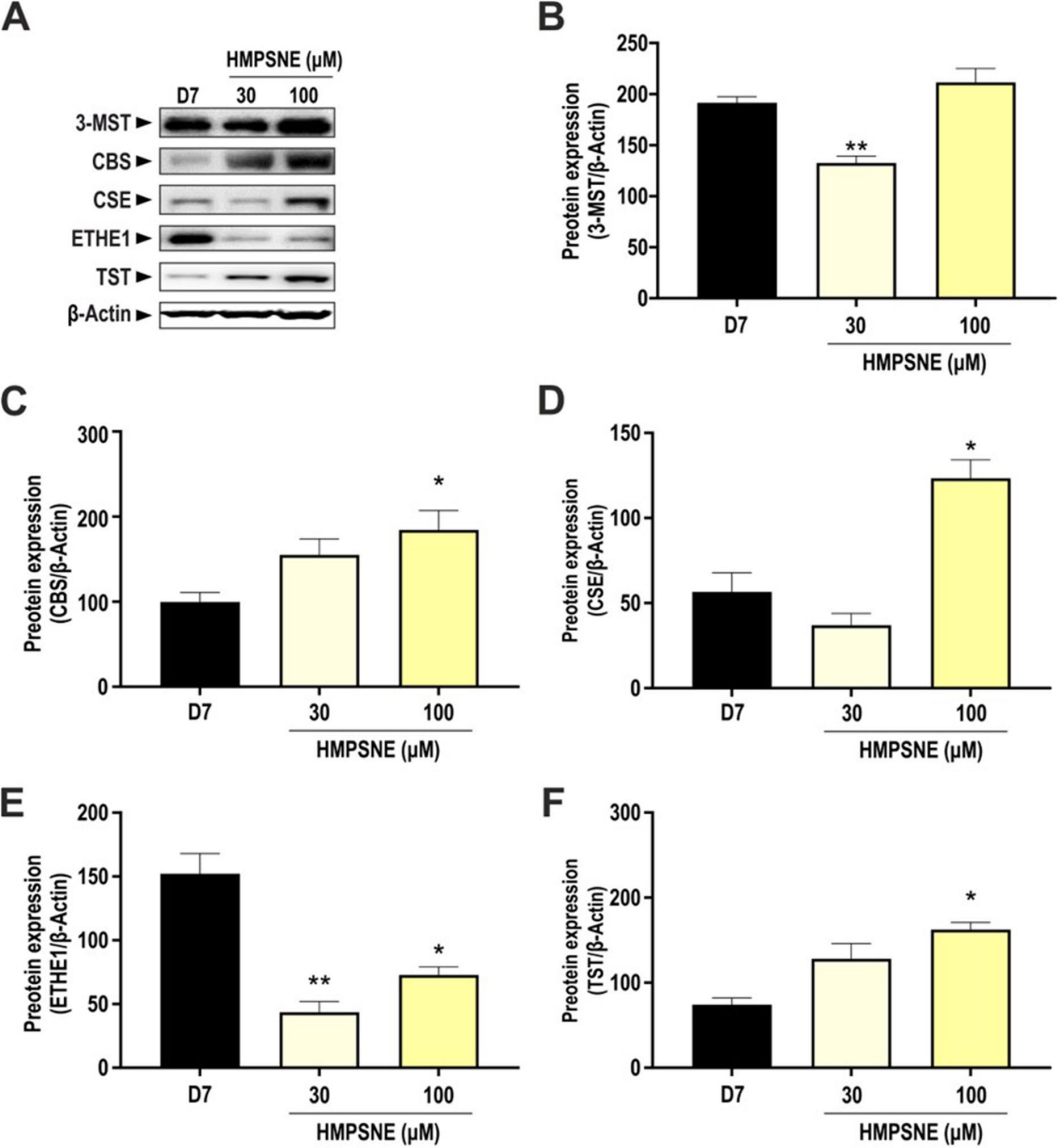
Expression of H_2_S-producing and -catabolizing enzymes in HMPSNE-treated cells. **A** Representative Western blot images of adipocytes treated with different concentration of HMPSNE and corresponding densitometry analysis of **B** 3-MST, **C** CBS, **D** CSE, **E** ETHE-1, and **F** TST in differentiated adipocytes (day 7). β-Actin was used as loading control. Data refer to mean values of *N* = 5 independent experiments ± SEM. *p < 0.05 and **p < 0.01 show significant differences compared to control values in the absence of HMPSNE treatment

Treatment with the 3-MST inhibitor HMPSNE did not have any marked or consistent effect on 3-MST protein expression; a slight inhibition was seen at 30 μM, and no effect was found at 100 µM (Fig. 5A, B). However, 100 µM HMPSNE significantly increased CBS (Fig. 5C), CSE (Fig. 5D), and TST (Fig. 5F) expression. ETHE-1 protein expression was markedly decreased in HMPSNE-treated adipocytes (Fig. 5E). These results showed that the pharmacological inhibition of 3-MST stimulates the expression of H_2_S-producing enzymes in adipocytes, possibly as a compensatory response to the inhibition of the production of 3-MST-derived H_2_S.

### Effect of GYY4137 or HMPSNE on intracellular H_2_S levels in differentiated adipocytes

**Fig. 6:**
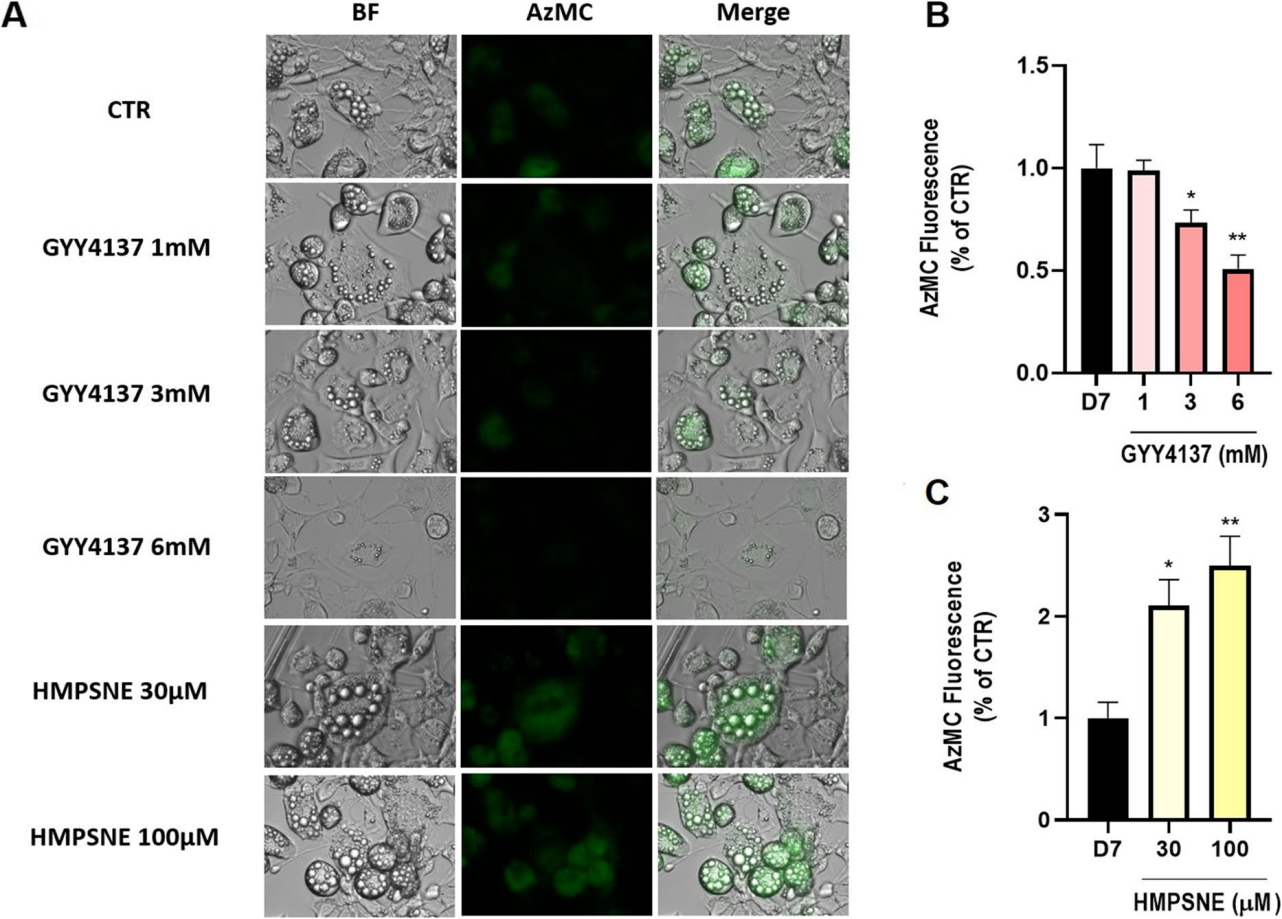
Quantification of cellular H_2_S content of differentiated adipocytes using the AzMC probe. **A** Bright field (BF) and AzMC fluorescence emitted by mature adipocytes (day 7). Quantification of AzMC signal at increasing concentrations of **B** GYY4137 and **C** HMPSNE. Data refer to mean values of *N* = 5 independent experiments ± SEM. *p < 0.05, **p < 0.01 show significant differences in AzMC fluorescence compared to control values in the absence of pharmacological modulator treatment

The detection of intracellular H_2_S in adipocytes treated with GYY4137 or HMPSNE was performed using cell live imaging in the presence of AzMC, a fluorogenic probe in which the aromatic azide moiety is selectively reduced in the presence of H_2_S, producing the fluorescent 7-amino-4-methylcoumarin with a concomitant increase in fluorescence (Fig. 6A). Unexpectedly, the cellular H_2_S signal was significantly reduced in GYY4137-treated adipocytes (Fig. 6B). In contrast, HMPSNE induced a concentration-dependent increase of H_2_S signal (Fig. 6C). We interpret the increase of cellular H_2_S signal in the HMPSNE-treated (Fig. 6B) cells as a consequence of the upregulation of H_2_S-producing enzymes, such as CBS and CSE (Fig. 5C, D). CSE has been previously reported to play a key role in the regulation of the adipogenesis, although with opposite effects as compared with 3-MST. Indeed, while CSE inhibition reduced lipid storage [10, 27], 3-MST inhibition increased lipid accumulation as shown both in vivo model [28] and in vitro (Fig. 2D, E). These observations suggest that 3-MST and CSE, in line with their different cellular localizations, have a different role in adipogenesis, thus pointing out that the endogenous H_2_S exerts different roles according to its biological source. Moreover, in contrast to CSE, the main product of the enzymatic activity of 3-MST is not H_2_S but sulfane sulfurs (polysulfides), which have been shown to have not always biological effects overlapping with those exerted by H_2_S [29].

Consistent with the observation done with Oil Red O staining, we also observed that the treatment of the cells with increasing concentration of GYY4137 was associated with a reduced number and size of lipidic drops (Fig. 6A). On the other hand, inhibition of 3-MST resulted in an increase of the adipocyte surface area, accompanied with an increase of the number of lipidic drops (Fig. 6A). Importantly, in a recently published study, increased adipocyte surface area was also demonstrated in vivo after 3-MST inhibition or silencing in C57Bl/6 J mice [28].

### Pharmacological inhibition of 3-MST induces multiple transcription factors involved in the regulation of adipogenesis

**Fig. 7:**
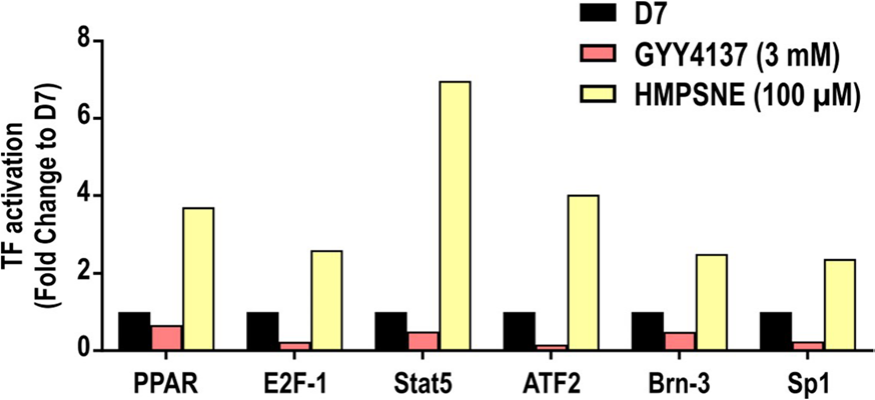
Effect of GYY4137 and HMPSNE treatments on transcription factor activation. Transcription factor profile of mature adipocyte treated with GYY4137 (3 mM) or HMPSNE (100 µM) in differentiated adipocytes (analyzed on day 7). Mean values of *N* = 2 independent determinations are shown. Selected transcription factors (TFs) with a | Fold change |> 2 are shown and are considered different compared to the control. Control transcription values are set to 1

Using a transcription factor array, we compared untreated adipocytes and HMPSNE- and GYY4137-treated adipocytes on day 7, when control cells were in their fully differentiated stage. The transcription factors that were showing more than twofold difference between control and treated cells are shown in Fig. 7. Among these, PPAR, E2F-1, Stat5, ATF2, Brn-3, Sp1, TCF/LEF, CAR, and CBF displayed a pronounced activation in HMPSNE-treated cells, and displayed the opposite profile in GYY4137-treated cells.

PPAR [30], E2F-1 [31], ATF2 [32], and Stat5 [33] are all known to act as promoters of adipocyte differentiation, in line with the effect of HMPSNE. Brn-3 regulates insulin-stimulated glucose transport in adipocytes [34]. Sp-1 is a negative regulator of C/EBPα, but in mature adipocyte, coordinates with PPARγ in order to regulate adipocyte triglyceride lipase (Atgl) [35]. The results suggest that 3-MST tonically suppresses the activation of various transcription factors involved in adipocyte differentiation. When 3-MST is inhibited, this repression is lifted, and the transcription factors execute the differentiation process.

### 3-MST silencing promotes lipid accumulation during adipocyte differentiation

**Fig. 8:**
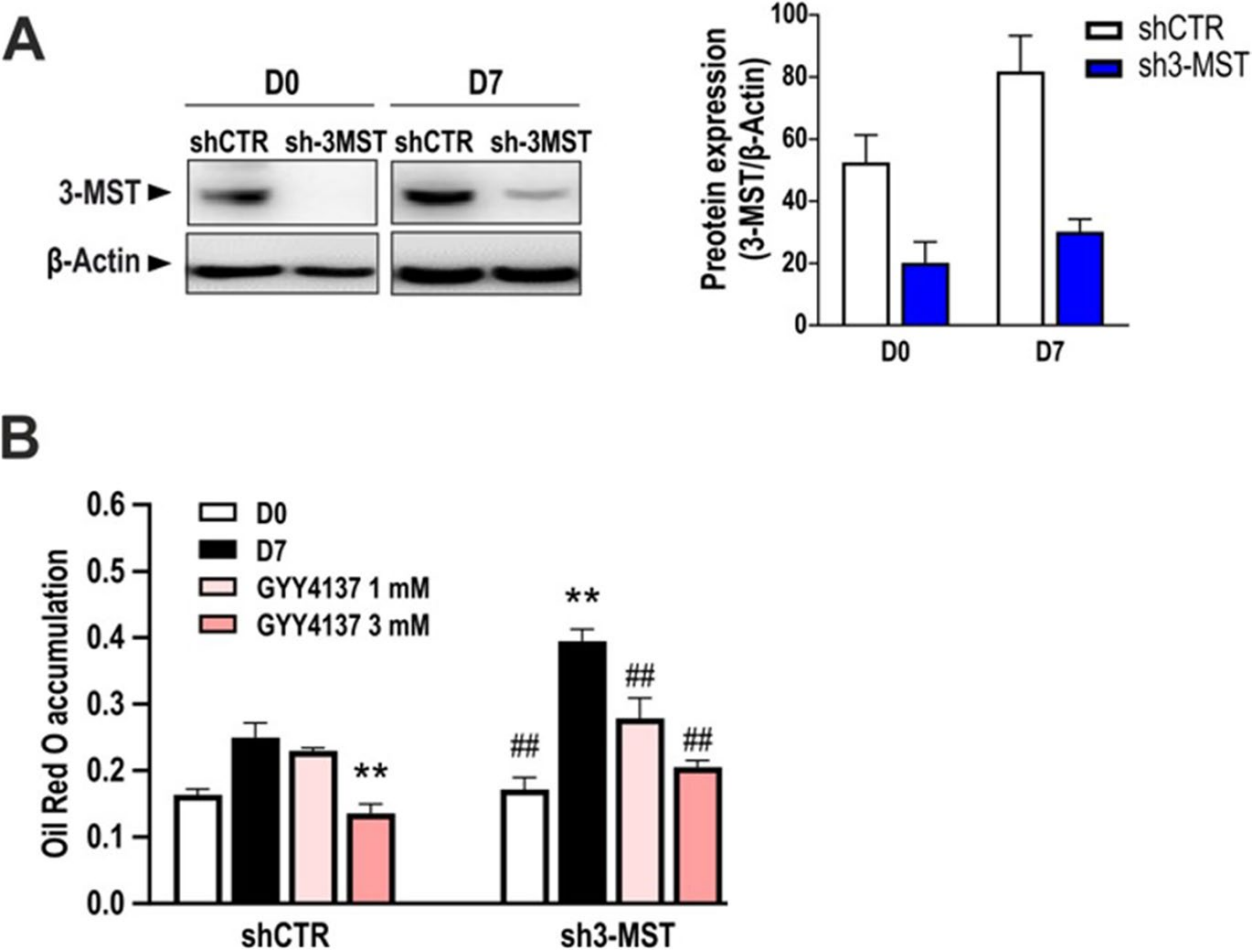
GYY4137 and HMPSNE treatments on 3-MST knock-down adipocytes. **A** Representative immunoblot showing the efficiency of 3-MST knockdown in pre-adipocytes (day 0) and mature adipocytes (day 7) and corresponding densitometry analysis. One-way ANOVA followed by Bonferroni post-test. *p < 0.05 compared to day 0 shCTR; #*p* < 0.05 compared to day 7 shCTR. **B** Oil Red O staining on control silenced (shCTR) or 3-MST silenced (sh3-MST) adipocytes treated with GYY4137 (1–3 mM). Data refer to mean values of *N* = 5 independent experiments ± SEM. *p < 0.05 and **p < 0.01 show significant differences in the measured values on day 7 (differentiated cells) compared to the initial values (non-differentiated values) on day 0; ^#^*p* < 0.05 and.^##^p < 0.01 show significant effect of 3-MST silencing or the H_2_S donor (GYY4137) compared to the corresponding control values

To further investigate the specific contribution of 3-MST on the process of lipid accumulation during adipogenesis, 3T3-L1 cells were stably transfected with shRNA against 3-MST, generating a 3-MST knockdown cell line (sh3-MST). Sh3-MST and control cells (shCTR) were differentiated into adipocytes, and the efficiency of 3-MST knockdown was confirmed by Western blotting (Fig. 8A). As noted previously [8, 10], the differentiation process increased the expression of 3-MST (Fig. 8A). Sh3-MST preadipocytes subjected to the standard adipocyte differentiation protocol (on day 7) displayed more pronounced lipid accumulation compared to shCTR, further suggesting that 3-MST has a biological role in negatively regulating adipocyte lipid uptake/cell differentiation in the current experimental model (Fig. 8A). This effect was reversed by co-administration of the H_2_S donor GYY4137 to the sh3-MST cells (Fig. 8B).

### Effect of GYY4237 or HMPSNE on cellular bioenergetics in mice adipose tissue

**Fig. 9:**
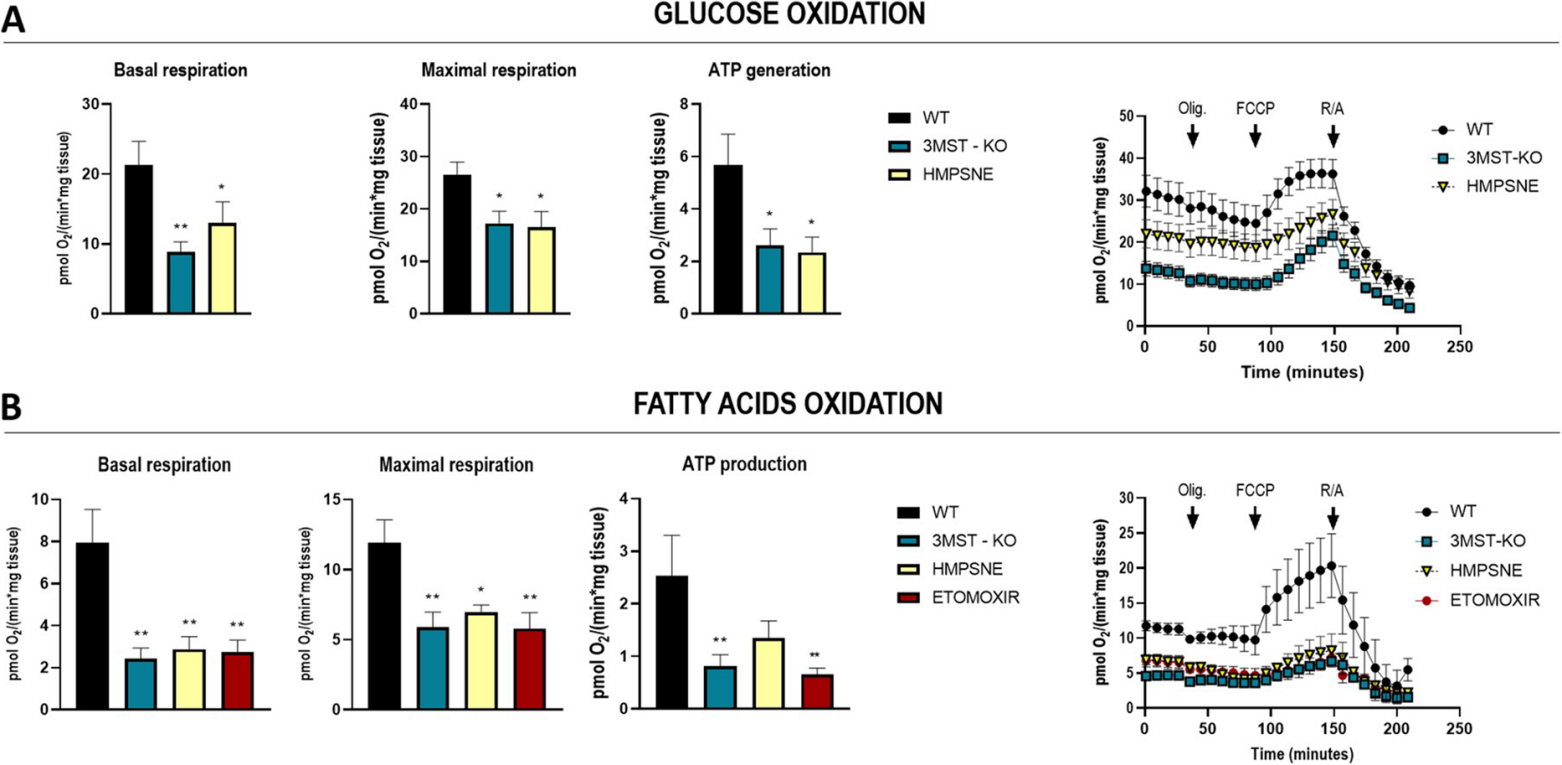
Effect of 3-MST pharmacological inhibition or knockout on bioenergetic parameters of inguinal white adipose tissue. Oxygen consumption rate measurements in inguinal white adipose tissue (iWAT) of WT and *Mpst*^*−/*^.^*−*^ mice in the presence of glucose (**A**) or palmitate (**B**) determined using a Seahorse Flux Analyzer. The assay protocol consisted of the subsequent addition of 50 µM oligomycin, 25 µM FCCP, and 20 µM rotenone/antimycin A, as described in the “Methods” section. Data refer to mean values of at least *N* = 5 independent experiments ± SEM. *p < 0.05 and **p < 0.01 show significant differences

Given the mitochondrial localization of 3-MST, we hypothesized that the modulation of 3-MST biochemical pathway could have an impact on mitochondrial respiration. Therefore, to evaluate the consequences of 3-MST pharmacological inhibition or its ablation, we carried out extracellular flux analysis using inguinal white adipose tissue (iWAT) collected from C57Bl/6 J mice or 3-MST knockout mice (*Mpst*^−/−^), as described in the “Materials and methods” section. Oxygen consumption rate (OCR) on iWAT tissues were assessed in the presence of either glucose or fatty acids (palmitate) as fuels to drive cellular bioenergetics (Fig. 9A, B). All the bioenergetic parameters, namely basal respiration, maximal respiration, and ATP production, were significantly lower in the WT tissues pre-treated with HMPSNE and *Mpst*^−/−^ mice as compared to WT (Fig. 9A, B). Particularly, in the fatty acid oxidation assay (FAO), the OCR of both HMPSNE-treated or *Mpst*^−/−^ iWAT recapitulated the decrease observed in the presence of the carnitine palmitoyltransferase inhibitor etomoxir (Fig. 9B). The mitochondrial impairment observed in *Mpst*^−*/*−^ mice as well as HMPSNE-treated tissues led overall to a reduced energetic metabolism, and in particular, the significantly reduced fatty acid oxidation may account for increased lipid storage.

## Discussion

During adipocyte differentiation, various H_2_S-producing enzyme systems become upregulated. However—in contrast to prior data focusing on the role of CSE and SELENBP1 (which act as endogenous *promoters* adipocyte differentiation) [8, 10]—the current study demonstrates that 3-MST acts as an endogenous *inhibitor* of the process of adipocyte lipid uptake during adipocyte differentiation. A similar role of 3-MST has also been observed in human dermal fibroblasts, where 3-MST silencing was found to promote adipogenic trans-differentiation [36]. In contrast, in isolated human preadipocytes, 3-MST knockdown led to significantly decreased expression of adipogenic, lipogenic, and insulin pathway–related genes suggesting a suppression of adipocyte differentiation [16]. Whether CBS serves a role as an endogenous suppressor or an endogenous enhancer of adipocyte differentiation appears to be dependent on the experimental model used; in vitro, CBS inhibition was found to enhance adipocyte differentiation [10, 16]; however, in vivo, CBS^−/−^ mice exhibit weight loss and have less adipose tissue than wild-type counterparts [37, 38].

The different role of various H_2_S-producing enzymes in the regulation of adipogenesis may be related to their unique localization and/or different cellular effectors. For example, 3-MST is partially mitochondrial, while CSE and CBS are largely cytosolic. Moreover, 3-MST—in contrast to CBS and CSE—produces large amounts of polysulfides, which exert their effects, to a significant degree through post-translational protein modifications (e.g., sulfhydration) [3, 5].

Several sets of data, showing that 3-MST expression decreases during adiposity [16, 19], suggest a functional role of 3-MST as an endogenous, tonic suppressor of adipocyte development: in the absence of functional 3-MST, the process of adipocyte becomes accelerated. Based on the data presented in the current report, the mechanisms by which endogenous 3-MST tonically suppresses adipocyte differentiation may be related to the regulation of various transcription factors involved in the differentiation process along with stimulation of the mitochondrial respiration and fatty acid oxidation. Interestingly, according to recent data, 3-MST and its enzymatic products H_2_S and polysulfide may post-transcriptionally modulate many effectors of adipocyte differentiation, including proteins involved in fatty acid and lipid metabolism, the citrate cycle, insulin signaling, various adipokines, and PPAR [16].

**Fig. 10:**
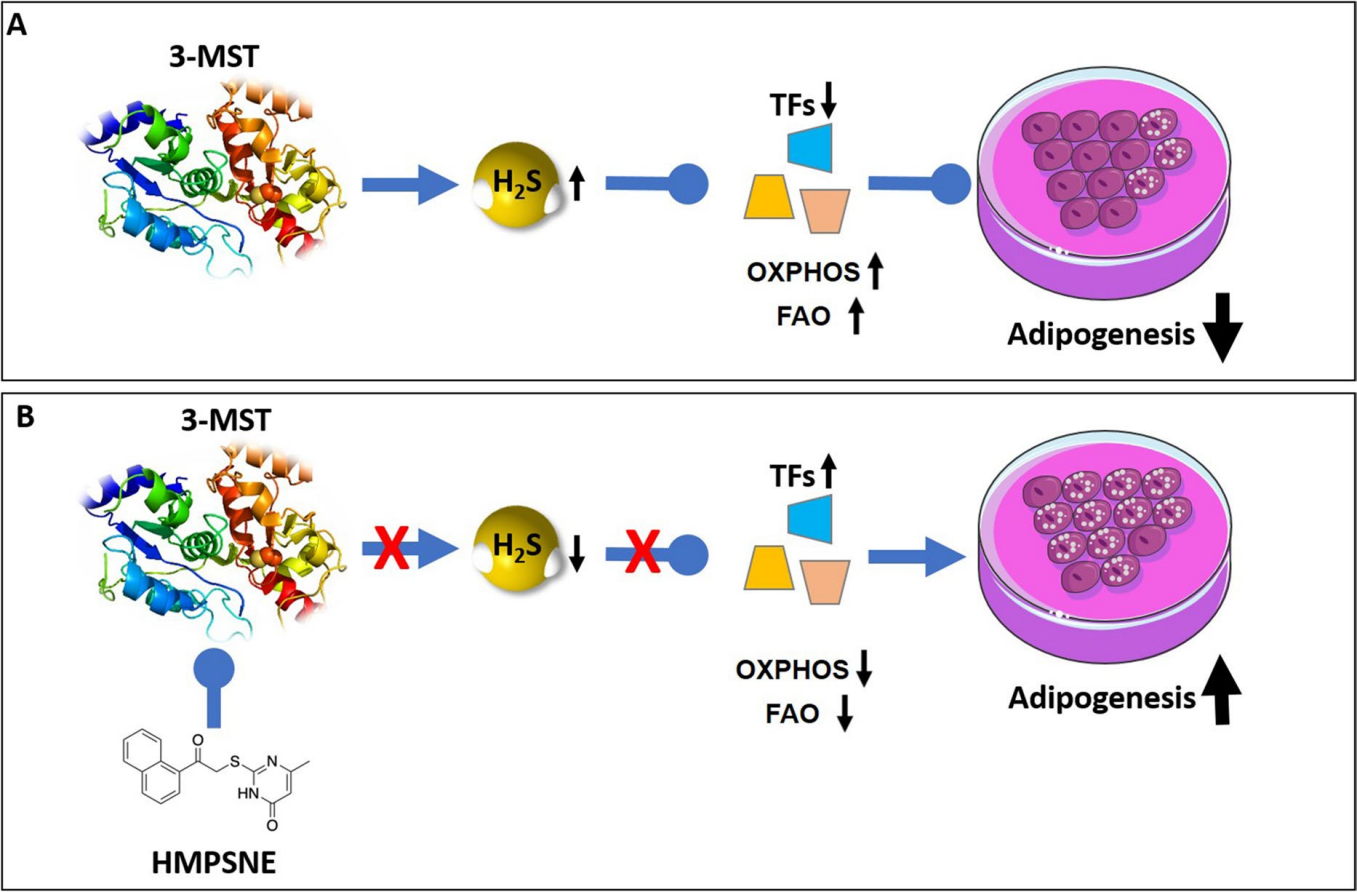
Role of 3-MST and the effect of HMPSNE on adipogenesis.** A** Under physiological conditions, 3-MST-derived H_2_S (and/or polysulfides, not shown) suppress the activation of various transcription factors that would normally stimulate adipogenesis, while sustaining oxidative phosphorylation (OXPHOS) and fatty acids oxidation (FAO). Via this mechanism the 3-MST system exerts a tonic suppressive effect on adipogenesis. **B** After 3-MST is inhibited—e.g., in the context of physiological aging—the tonic inhibition of transcription factors becomes disinhibited. In turn, these factors, according to their normal biological role, promote adipogenesis

A recently published study has also explored some of the molecular mechanisms related to the regulation of lipogenesis by 3-MST using a murine high-fat-diet model [28]. This study concluded that 3-MST ablation leads to increase of ROS levels with consequent stimulation of a cascade of events triggered by HIF1α activation. The same study also demonstrated that HIF1α, in turn, downregulates the expression levels of translocase of inner/outer mitochondrial membrane (in TIM/TOM complex), thus impairing mitochondrial protein import. This effect has various functional consequences, including suppression of Krebs cycle, oxidative phosphorylation, and fatty acid oxidation, eventually leading to excessive lipid accumulation, increased iWAT mass, impaired glucose/insulin tolerance, and increased body weight [28]. In the current paper as well as in the in vivo study discussed in the current paragraph [28], H_2_S administration—by means of either the slow releaser GYY4137 (current study) or the donor compound SG1002 [28]—reversed the chain of events leading to lipid accumulation both in vitro and in vivo. Indeed, several lines of independent, emerging data [16, 18, 19] suggest that the 3-MST/H_2_S system serves as a counterregulatory system to tonically inhibit adipocyte development, and, consequently, obesity (Fig. 10). Multiple studies show that H_2_S levels decline during aging [6, 7]. The underlying mechanisms are multiple, and it is logical to hypothesize that aging—at least in part via inhibition of the 3-MST pathway—may, in fact, sensitize to adipocyte fat accumulation and may promote the development of obesity.

Already in 1984, Finkelstein and Benevenga observed that production of H_2_S and other “volatile sulfur compounds” showed a gradual decreased with increasing age [40]. This decline has been since observed in several different animal species, as well as in humans [6, 7, 39–42]. The decline applies to total sulfane sulfur compounds’ plasma levels, and the amount of sulfhydrated proteins shows age-dependent declines that mirror the above-discussed decreases in free H_2_S levels. The decrease in protein sulfhydration, in turn, “gives way” to oxidative protein modifications, characterized by the formation of sulfonylation of protein cysteines [43].

The underlying mechanisms, as far as the expression or activity of various H_2_S-generating enzymes are concerned, are only partially understood, but it appears that in many—but not all—studies, CBS and CSE expression declines with age [6, 42–45]. This downregulation may be, at least in part, due to ablation of the transcriptional regulation of CSE and CBS expression by thyroid hormone signaling [45, 46], although multiple other processes may also be involved; for instance, the increased reactive oxygen species “burden” that occurs during aging may lead to an increased oxidative “consumption” of H_2_S [7]; a similar phenomenon has been also demonstrated in the context of diabetes-associated vascular dysfunction [47].

Because different H_2_S-producing enzymes can have different biological roles, most relevant for our study are the changes in the 3-MST pathway associated with aging (and with obesity). 3-MST expression shows an age-dependent decrease in the heart and kidney of rats and mice [48–50]. Moreover, the bioenergetic stimulatory effect of 3-mercaptopyruvate (the substrate of 3-MST) was markedly lower in liver mitochondria isolated from aged mice, when compared to the marked stimulating effect seen in mitochondria isolated from young mice [51]. Since 3-MST is subject to oxidative inactivation [52, 53], we hypothesize that during aging, the loss of 3-MST-associated biological functions may be due to a combination of transcriptional effects (downregulation of 3-MST mRNA and protein expression) and posttranscriptional effects (oxidative inactivation of the enzyme due to aging-associated increase in oxidative stress). Similarly, adiposity, on its own, has been shown to downregulate 3-MST expression [19]. Importantly—while young adult 3-MST^−/−^ mice do not show a characteristic phenotype—18-month-old 3-MST^−/−^ mice exhibit a hypertensive phenotype and cardiac hypertrophy [54], as well as increased fat accumulation when placed on high-fat diet [28], underlying the importance of the 3-MST pathway in counteracting the development of various age-associated processes.

## Perspectives

The current study, as well as recently emerging data linking the 3-MST pathway to obesity and various age-associated diseases (see above), should stimulate further research in the field aimed at the restoration of the H_2_S balance during aging. In general, there are already a large number of studies—from *C. elegans* model system to rodents—that support the general concept that H_2_S donation may be an “anti-aging” approach [6, 7, 39, 46]. Nevertheless, several important issues remain to be further investigated, including the consideration of potential sex/gender differences in the H_2_S-related metabolic and other regulatory processes discussed above. While there are significant sex differences in various H_2_S pathways and processes [6], these differences have not yet been investigated in the context of aging; this is one of the areas which remains to be explored in the future.

What, then, are our experimental therapeutic options to compensate the decline of the 3-MST pathway in adiposity and aging-associated diseases? The first group of approaches may focus on preventing the downregulation and/or oxidative inhibition of 3-MST during aging. Some of the most obvious approaches to achieve this goal may involve exercise—which is known to counteract aging-associated decline in 3-MST mRNA expression in the heart [49]—or various dietary interventions, including various intermittent fasting or other dietary restriction approaches [39, 46]. Betaine, [55] as well as pyridoxal-5-phosphate [56], has also been recently shown to upregulate 3-MST expression in the brain and heart of rodents, respectively. There may be also pharmacological possibilities for a redox-based “reactivation” of 3-MST [53, 54, 57].

Another approach may be “H_2_S donation,” either by administering the substrate of 3-MST, 3-mercaptopyruvate [53, 57], or by using the more general “H_2_S donor administration” approach. There are many pharmacological avenues to achieve this latter goal; H_2_S donors with various release rates have been designed and tested, some of them organelle-targeted, some redox-triggered. These molecules are subject to focused review articles [5, 58–64]. Unfortunately, these approaches are currently only in the preclinical stage, with the notable exception of SG-1002, which is a clinical-stage molecule, and which exhibits protective effects in various preclinical models of aging-associated diseases including atherosclerosis, chronic heart failure, and diet-induced obesity [61, 65–68].

Taken together, the current study—in conjunction with multiple recent studies focusing on the therapeutic aspects of H_2_S biology—should stimulate further work and ultimate translational progress in the experimental therapy of various aging-associated diseases and conditions including obesity.

## Data Availability

The datasets during and/or analyzed during the current study available from the corresponding author on reasonable request.

## Abbreviations

3-MP: 3-Mercaptopyruvate
3-MST: 3-Mercaptopyruvate sulfurtransferase
ATCC: American Type Culture Collection
ATP: Adenosine triphosphate
BSA: Bovine serum albumin
CBS: Cystathionine-β-synthase
CSE: Cystathionine-γ-lyase
DMSO: Dimethyl sulfoxide
ETHE1: Ethylmalonic encephalopathy 1 protein
FBS: Fetal bovine serum
FCCP: Carbonyl cyanide 4-(trifluoromethoxy)phenylhydrazone
H_2_S: Hydrogen sulfide
HIF1α: Hypoxia-inducible factor 1-alpha
HMPSNE: 2-[(4-Hydroxy-6-methylpyrimidin-2-yl)sulfanyl]-1-(naphthalen-1-yl)ethan-1-one
HRP: Horseradish peroxidase
IgG: Immunoglobulin G
iWAT: Inguinal white adipose tissue
LDH: Lactate dehydrogenase
MTT: 3-(4,5-Dimethylthiazol-2-yl)-2,5-diphenyltetrazolium bromide
OCR: Oxygen consumption rate
PBS: Phosphate-buffered saline
SDS: Sodium dodecyl sulfate
SEM: Standard error of the mean
TF: Transcription factor
TST: Thiosulfate sulfurtransferase
